# *Quasitagalis afonsoi*, a new genus and a new species of Saicinae (Hemiptera, Reduviidae) inhabiting a cave in Brazil, with an updated key to the genera of Saicinae of the New World

**DOI:** 10.3897/zookeys.966.52930

**Published:** 2020-09-09

**Authors:** Hélcio R. Gil-Santana, Jader Oliveira, Robson de A. Zampaulo

**Affiliations:** 1 Laboratório de Diptera, Instituto Oswaldo Cruz, Av. Brasil, 4365, 21040-360, Rio de Janeiro, RJ, Brazil Instituto Oswaldo Cruz Rio de Janeiro Brazil; 2 Laboratório de Parasitologia, Universidade Estadual Paulista “Julio de Mesquita Filho”, Faculdade de Ciências Farmacêuticas UNESP/FCFAR, Rodovia Araraquara Jaú, KM 1, 14801-902, Araraquara, SP, Brazil Universidade Estadual Paulista “Julio de Mesquita Filho” Araraquara Brazil; 3 VALE SA, Gerência de Licenciamento e Espeleologia (COI), Av. de Ligação, 3580, prédio 1, 1° andar, Mina de Águas Claras, 34000000, Nova Lima, MG, Brazil VALE SA, Gerência de Licenciamento e Espeleologia Nova Lima Brazil

**Keywords:** Heteroptera, male genitalia, Neotropics, *
Paratagalis
*, *
Tagalis
*

## Abstract

*Quasitagalis afonsoi***gen. et sp. nov.** of Saicinae (Hemiptera, Reduviidae) is described based on a male and three female specimens collected in a cave in the State of Tocantins, Brazil. Additionally, some characteristics from two nymphs of different instars of the same species are also recorded. An updated key to the New World genera of Saicinae is provided.

## Introduction

There are ten genera of Saicinae in the Neotropical region, five of which are currently monotypic (Gil-Santana et al. 2015). A summary of the taxonomy of this group and a key to the genera of the New World were provided by Gil-Santana et al. (2015).

Little is known of the biology and natural history of Saicinae (Gil-Santana et al. 2010). Specimens have been most commonly collected at lights (Schuh and Weirauch 2020) or swept and beaten from vegetation (Gil-Santana et al. 2015). Gil-Santana et al. (2010) included a synopsis of the biological and ecological information available for New World Saicinae, recording *Tagalis evavilmae* Gil-Santana, Gouveia & Zeraik, 2010 as an inhabitant of birds’ nests, a first for Saicinae.

Among Neotropical Saicinae, sexual dimorphism consisting of larger eyes and longer, ciliated setae on the first antennal segment of males was observed in *Paratagalis spinosus* Monte, 1943 (Gil-Santana and Costa 2009), and it was confirmed in the following species of *Tagalis* Stål, 1860: *T. evavilmae*, and *T. seminigra* Champion, 1899 (Gil-Santana et al. 2010). In *T. inornata inornata* Stål, 1860, however, the male eyes were shown not to be much larger than those of females and the long ciliated setae on the first antennal segments were shorter than those of some other species (Gil-Santana 2011). Large eyes and long ciliated setae on the first antennal segments of males have also been recorded in the following species of *Tagalis*: *T. baenai* Gil-Santana, 2011, *T. grossii* Gil-Santana, 2011, *T. marquesi* Gil-Santana, 2011 (Gil-Santana 2011), *T. dichroa* Castro-Huertas & Forero, 2014 (Castro-Huertas and Forero 2014) and *T. drakkar* Varela & Melo, 2017 (Varela and Melo 2017). However, since no females of these species are known, the possible sexual dimorphism could not be verified. Longer ciliated setae on the first third of the second antennal segment of males was also observed in *P. spinosus* (Gil-Santana and Costa 2009), while they were recorded on both the first and the second antennal segments in males of the species of *Oncerotrachelus* Stål, 1868 studied by Gil-Santana (2013).

On the other hand, McAtee and Malloch (1923) recorded that the length of the spines on the fore femora for *Tagalis* was sexually dimorphic and, to some extent, also exhibited intraspecific variation, and therefore would not seem to be of taxonomic importance. The armature of the fore femora in *T. i. inornata* was shown to have smaller spines in males when compared to those of the females (Gil-Santana 2011). However, the apparent sexual variation in length of the spines of the fore femora (McAtee and Malloch 1923) may be related to the size of the individual, since the females are usually larger. This characteristic and individual variation would be better evaluated through examination of more specimens, including other species.

A scopula, previously documented for some taxa of Emesinae (Wygodzinsky 1966), was recently recorded in several Saicinae (Weirauch and Forero 2007a; Weirauch 2007, 2008a). In Saicinae, the scopula is present as a hairy attached structure on the ventral surface of the apex of the third tarsomere of all pairs of legs. It was recorded in several species of Saicinae (Weirauch 2007), among which only two were from the New World, *Saica recurvata* (Fabricius, 1803) (Weirauch 2007) and *Kiskeyana palassaina* Weirauch & Forero, 2007 (Weirauch and Forero 2007a, b). Later, Castro-Huertas and Forero (2014) recorded the presence of the scopula on the apex of the third tarsomere in all the legs on both species of *Tagalis* described by them. Regarding its function, Weirauch and Forero (2007a) speculated that the scopula might be a structure that assists in movement on smooth (e.g., plant) surfaces, while Weirauch (2007) although arguing that the scopula in Saicinae could be exclusively used for locomotion or that it could play a certain role in prey capture, concluded that its primary function is probably during locomotion. Yet, it was considered as a synapomorphy of part of the Saicinae by Weirauch (2008a).

Our knowledge of immature stages of Saicinae is very limited (Rédei 2004). Among the Saicinae from the New World, nymphal stages have only been described in one species, *Tagalis evavilmae* (Gil-Santana et al. 2010). On one hand, the latter authors recorded that the nymphs of *T. evavilmae* show common features found in immature Heteroptera, such as a bi-segmented tarsi and smaller eyes in younger instars (Schuh and Weirauch 2020; Rédei 2004). On the other hand, Gil-Santana et al. (2010) also recorded the presence of different patterns of features with taxonomical significance in nymphs when compared with adults (like an additional spine on the ventral side of the head, more than three spines on tibiae) and argued that these characteristics which are observed in adults of other related genera, might help to understand the relationships among the genera of Saicinae in future studies.

*Quasitagalis afonsoi* gen. et sp. nov. of Saicinae (Hemiptera, Reduviidae) is described based on a male and three female specimens collected in a cave in the State of Tocantins, Brazil. Additionally, some characteristics from two nymphs of different instars of the same species are recorded too. An updated key to the New World genera of Saicinae is provided.

## Materials and methods

All fieldwork, including the collection of the specimens inside a cave, was undertaken by the third author (RAZ), who also provided Figs 60–63.

**Figures 1–3.**
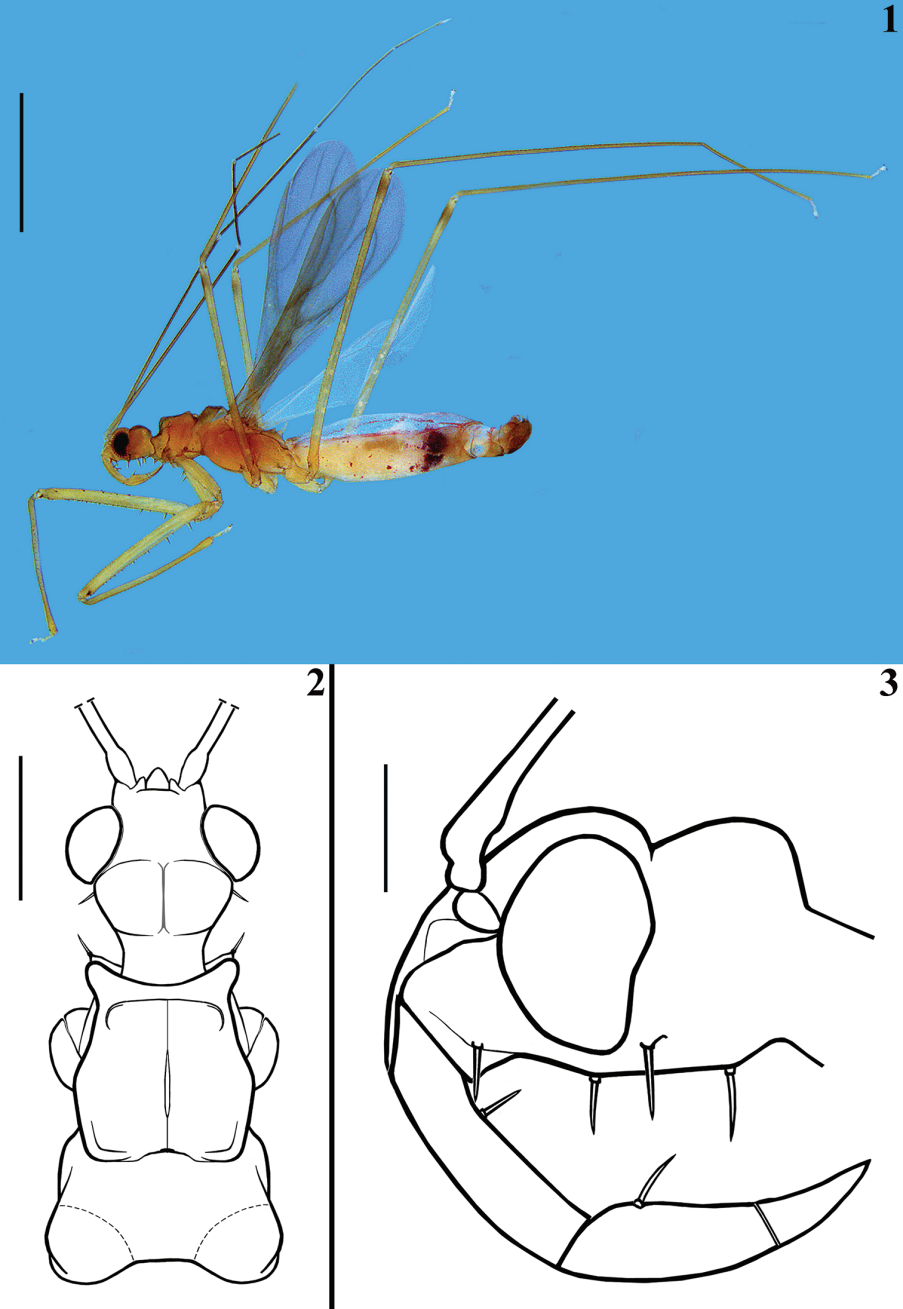
*Quasitagalis afonsoi* gen. et sp. nov., male holotype **1** lateral view **2** head and pronotum, dorsal view **3** head, lateral view. Scale bars: 2.0 mm (**1**); 0.5 mm (**2**); 0.2 mm (**3**).

Photographs of the male holotype and a female paratype of *Quasitagalis afonsoi* gen. et sp. nov. (Figs 1, 21) were taken by João Paulo Sales Oliveira Correia (“Laboratório Nacional e Internacional de Referência em Taxonomia de Triatomíneos” (LNIRTT), Instituto Oswaldo Cruz (IOC), Rio de Janeiro, Brazil), with a Leica DMC 2900 camera attached to a Leica M205C stereomicroscope. Several images were stacked using the LAs software version 4.9.

Scanning electron microscopy images (Figs 23–28, 31, 32, 38, 40–43, 45–59, 64, 65) were obtained by the second author (JO). A female, two nymphs of different instars of the new species, and a female of *Tagalis inornata inornata* Stål, 1860 were cleaned in an ultrasound machine. Subsequently, the samples were dehydrated in alcohol, dried in an incubator at 45 °C for 20 min, and fixed in small aluminium cylinders with transparent glaze. Sputtering metallisation was then performed on the samples for 2 min at 10 mA in an Edwards sputter coater. After this process, the samples were studied and photographed using a high-resolution field emission gun scanning electron microscope (FEG-SEM; JEOL, JSM-7500F), similarly as described by Rosa et al. (2010, 2014).

**Figures 4–7.**
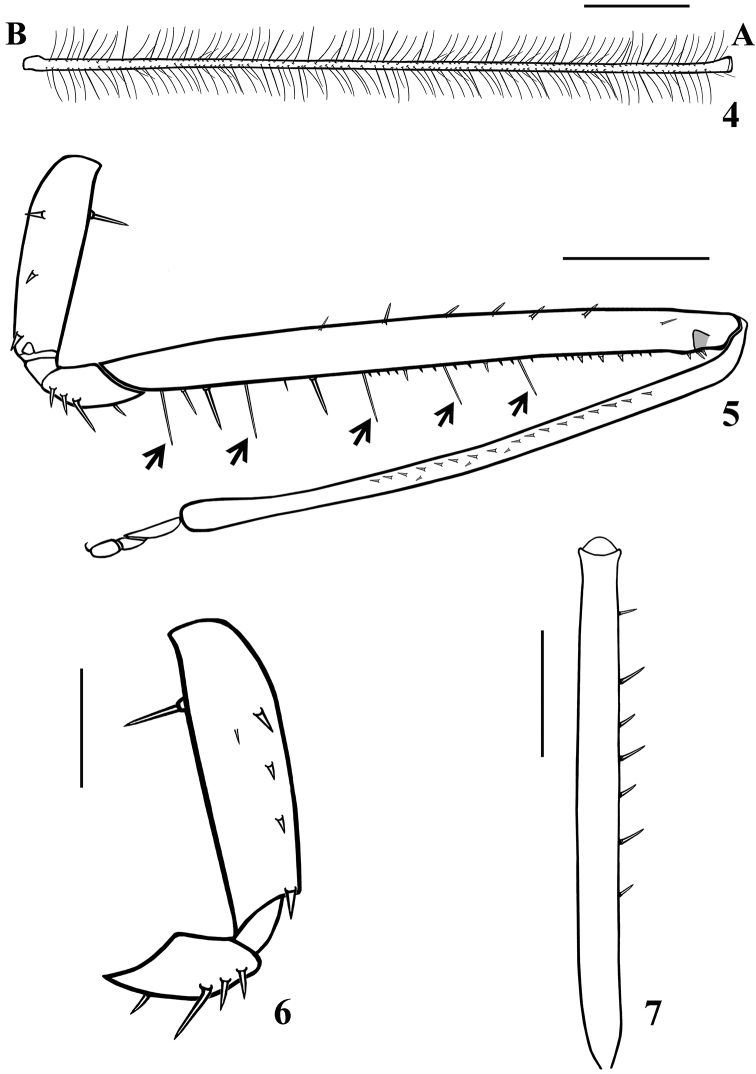
*Quasitagalis afonsoi* gen. et sp. nov., male holotype **4** first antennal segment (general vestiture omitted, except the ciliated setae) (**A** apex, **B** base) **5** left fore leg, lateral view, inner face (setae omitted, except the longer setae, pointed by arrows) **6** right fore coxa and trochanter, lateral view, inner face **7** left fore femur, dorsal view. Scale bars: 0.5 mm (**4, 5, 7**); 0.2 mm (**6**).

All remaining figures were produced by the first author (HRG-S). The fixed adults, microscopic preparations, and genitalia were photographed using a digital camera (Sony DSC-W830). Drawings were made using a camera lucida. For clarity, the general vestiture (setation) in several ink drawings was completely (Figs 2, 3, 6, 7, 11, 22, 33, 34, and 44) or almost completely (Figs 4, 5, and 39) omitted. Images were edited using Adobe Photoshop CS6. Dissections of the male genitalia were made by first removing the pygophore from the abdomen with a pair of forceps and then clearing it in 20% NaOH solution for 24 hours. The dissected structures were studied and photographed or drawn in glycerol.

Observations were made using a stereoscope microscope (Zeiss Stemi) and a compound microscope (Leica CME). Measurements were made using a micrometer eyepiece. General morphological terminology mainly follows Lent and Wygodzinsky (1979) and Schuh and Weirauch (2020). The (visible) segments of labium are numbered as II to IV, given that the first segment is lost or fused to the head capsule in Reduviidae (Weirauch 2008b, Schuh et al. 2009). Gil-Santana et al. (2010) and Gil-Santana (2011) were mostly followed in the case of the terms applied to structures that are characteristic or peculiar to Saicinae.

The holotype and two female paratypes will be deposited in the Entomological Collection of the “Museu Nacional da Universidade Federal do Rio de Janeiro”, Rio de Janeiro, Brazil (MNRJ) and the female paratype, the nymphs and a female of *Tagalis i. inornata* used for obtain SEM images were deposited in the Dr Jose Maria Soares Barata Triatominae Collection (CTJMSB) of the São Paulo State University Julio de Mesquita Filho, School of Pharmaceutical Sciences, Araraquara, São Paulo, Brazil. All measurements are in millimetres (mm).

## Results

### Taxonomy

#### Subfamily Saicinae

##### Quasitagalis gen. nov.

Taxon classificationAnimaliaHemipteraReduviidae

44FD9214-1435-5A0E-B31C-5133F92CE197

http://zoobank.org/1549BE8A-08FE-455F-A9BB-FB42A97E373A

###### Type species.

*Quasitagalis afonsoi* sp. nov., by present designation.

###### Diagnosis.

*Quasitagalis* gen. nov. can be separated from other genera of Saicinae of the New World by the combination of the characters presented in the key below; among them, *Quasitagalis* gen. nov. seems to be closer to *Tagalis*. However, these two genera can be promptly separated by the following set of characters: ventral portion of the head (gula) with a distal pair of strong setigerous spines, posterior to the eyes, in both genera, while only in *Quasitagalis* gen. nov., another pair is present below (between) the eyes; scutellum tapering into an erect spine in *Tagalis* and slightly elevated, subtriangular, without a spine in *Quasitagalis* gen. nov.; and inner surface of fore tibia with three or four (*T. femorata* Melo, 2008) very strong setigerous spines implanted close to dorsal surface (*Tagalis*) or with a simple (male) or double (female) longitudinal median row of numerous shorter spines (*Quasitagalis*).

###### Description.

Adults. ***Head***: transversal sulcus deep, reaching eyes at hind margin; postocular portion subglobose, faintly depressed at median portion. Eyes globose in dorsal view, suboval in lateral view; strong setigerous spines anteroventrally and posteroventrally from eyes, the former somewhat smaller than the latter and ventrally, on gula, two pairs of similar setigerous spines: one pair below (between) the eyes and other pair, posterior to the eyes, somewhat closer to the neck than to the eyes. Antennal segments slender; segment I longest, clothed with long fine (ciliated) setae in males; segment II and III longer than half or half as long as the first segment; segment IV approximately one third as long as the first segment. Labium: segment II slender, elongate, curved, almost reaching posterior margin of eyes, with a pair of stout spines slightly basal to midpoint; segment III swollen mainly in the first third, where another pair of stout spines are located; segment IV slender, tapering. ***Thorax***: prothorax divided by a transverse deep furrow between fore and hind lobes of pronotum, interrupted at median portion, above which there is a deep small excavation; anterolateral angles prominent as rounded tubercles; fore lobe subquadrate with pairs of lateral somewhat acute dorsal swellings or humps anteriorly and posteriorly, the latter more prominent; a longitudinal shallow and narrow median furrow, slightly larger at midportion, its posterior portion ending at the median deep excavation mentioned above; disc of fore lobe finely rugose; hind lobe trapezoidal, becoming larger to the posterior margin; integument more coarsely rugose on disc, which is slightly depressed; humeral angles rounded. Lateral shallow ridge reaching from tubercles of anterolateral angles towards posterior swellings of fore lobe, prominent at anterior half and shallower posteriorly. Scutellum base broad, apex slightly elevated, subtriangular, spineless. Metanotum with a short erect tubercle followed by a short obliquely erect spine larger at base and blunt at apex. Proepisternal processes projected with a pair of strong setigerous spines, anterodorsal spine moderately curved, posteroventral almost straight. Supracoxal lobes of propleura prominent. Prosternum larger on anterior margin; stridulitrum long, narrow. Mesosternum larger than prosternum and metasternum; meso and metasternum with a longitudinal, thin, shallow median keel. Fore legs stouter and shorter than others; fore coxa elongated, cylindrical, with a long spine on basal third of anterior surface, and three or five spines on inner face; mid and hind coxae ovoid; trochanters triangular, tapering; fore trochanter with four spines on inner side, three of which closer to each other at approximately basal half and the fourth spine variably set more or less apart and more ventrally at the apical half of the segment; fore femur stout, slightly curved in lateral view, armed ventrally with a few short spines and a variable number of few longer ones intermixed, a small subapical ventral protuberance with two or three small spines; a row of short spines on upper margin of inner surface; between the latter and the ventral line of spines, in the females, a row of somewhat more numerous, setigerous spines; fore tibia slightly curved in lateral view, apically expanded, with a single (male) or double (female) longitudinal median row of numerous spines running on approximately 1/2 (male) or 2/3 (female) of the median portion of inner surface. Middle and hind legs long and slender. Tarsi with three segments; first the longest; claws simple; scopula present on ventral portion of apex of third tarsomere of all legs. Forewings with two closed cells; distal cell much larger than basal one. ***Abdomen*** elongated, cylindrical in the male and ovoid in the female. In male, pygophore with a medial distal process; parameres symmetrical, short, apex rounded with an apical elongate acute spine acute at its centre.

###### Distribution.

Brazil, State of Tocantins.

###### Etymology.

The name of the new genus was composed by the Latin word *quasi*, meaning almost, nearly, like, and *Tagalis*, in reference to its apparent proximity to the latter genus. The gender is feminine.

##### Quasitagalis afonsoi sp. nov.

Taxon classificationAnimaliaHemipteraReduviidae

E3658BD9-6DD7-57AC-9FD4-4230804CB886

http://zoobank.org/8006DD5A-3868-4742-B152-86B2640437E2

Figures 1–3 , 4–7 , 8–13 , 14–17 , 18–20 , 21–24 , 25–32 , 33–37 , 38, 39 , 40–47 , 48–51

###### Type material.

**Brazil**, Tocantins, Lavandeira, Gruta da Gia [Gia’s Cave], 12°49'42"S, 46°20'43"W, 05–10.i.2009, Robson A. Zampaulo leg.: ***Holotype*** (male), 2 ***Paratypes*** (females) (MNRJ), 1 ***Paratype*** (female), (CTJMSB, 861).

###### Description.

**Male**. Figures 1–20. Measurements are given in Table 1.

***Coloration.*** General coloration testaceous; approximately distal half of first antennal segment and the other antennal segments darkened; articulations between the segments and extreme apex of antennal segment IV pale; femora somewhat paler, the fore pair even more; fore femora with a small subapical pair of lateral dark spots on inner and outer surfaces just distal or above a small spiny protuberance; middle and hind femora with a subapical faint darkened ring; tarsi pale to whitish; forewings greyish, with the veins slightly darkened; hind wings translucent, veins pale yellowish; abdomen with a reddish tinge on the connexivum and on adjacent portion of tergites; most abdominal segments paler, darkened to the apex, including the ventral visible portion of segment VIII and the genital capsule (Fig. 1).

**Table 1.** Measurements (mm) of adult types of *Quasitagalis afonsoi* gen. et sp. nov.

	Male holotype	Female paratypes (N = 3)		
		Maximum	Minimum	Mean
Body length to tip of forewing	6.00	6.40	6.00	6.20
Body length to tip of abdomen	5.50	6.20	5.70	5.90
Head length (excluding neck)	0.70	0.70	0.70	0.70
Anteocular portion length	0.20	0.30	0.20	0.23
Postocular portion length	0.20	0.30	0.20	0.25
Head width across eyes	0.60	0.60	0.60	0.60
Interocular distance (synthlipsis)	0.30	0.30	0.30	0.30
Transverse width of eye	0.20	0.20	0.10	0.17
Length of eye	0.25	0.25	0.20	0.23
Antennal segment I length	3.10	3.30	3.10	3.20
Antennal segment II length	1.80	2.00	1.90	1.93
Antennal segment III length	1.80	1.80	1.50	1.67
Antennal segment IV length	1.00	1.00	0.90	0.97
Labial segment II length	0.50	0.50	0.50	0.50
Labial segment III length	0.30	0.30	0.30	0.30
Labial segment IV length	0.20	0.25	0.20	0.21
Fore lobe of pronotum length	0.60	0.60	0.60	0.60
Fore lobe of pronotum max. width	0.65	0.70	0.65	0.68
Hind lobe of pronotum length	0.50	0.50	0.50	0.50
Fore lobe of pronotum max. width	0.90	1.00	0.90	0.97
Forewing length	4.10	4.70	4.00	4.37
Fore coxa length	0.80	0.80	0.70	0.76
Fore femur length	2.10	2.20	1.90	2.06
Fore tibia length	1.90	2.00	1.70	1.86
Fore tarsus length	0.30	0.30	0.30	0.30
Mid femur length	2.90	3.10	2.50	2.86
Mid tibia length	3.80	3.80	3.40	3.66
Mid tarsus length	0.25	0.25	0.25	0.25
Hind femur length	4.10	4.50	3.80	4.23
Hind tibia length	6.00	6.30	5.50	5.93
Hind tarsus length	0.25	0.30	0.20	0.23
Abdomen length	3.10	3.50	3.00	3.20
Abdomen maximum width	0.90	1.40	0.70	1.06

**Figures 8–13.**
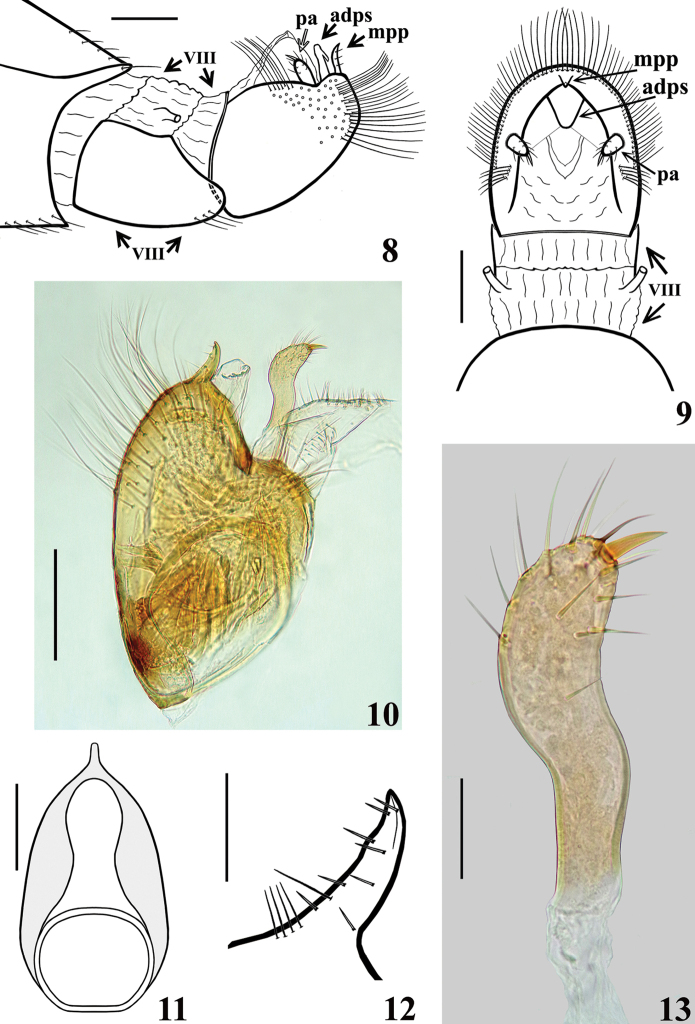
*Quasitagalis afonsoi* gen. et sp. nov., male holotype **8, 9** apical margin of abdominal segment VII, abdominal segment VIII and genital capsule **8** lateral view **9** dorsal view **10–13** male genitalia **10** pygophore and left paramere, lateral view **11** pygophore without parameres, dorsal view **12** medial process of pygophore, lateral view **13** right paramere. Abbreviations: adps apex of dorsal phallothecal sclerite, mpp medial process of pygophore, pa paramere, VIII abdominal segment VIII. Scale bars: 0.2 mm (**8–11**); 0.05 mm (**12, 13**).

***Vestiture.****Body* generally covered by sparse, thin, pale, suberect, obliquely erect or adpressed setae. *Head* with somewhat longer and more numerous setae on anterior portion, clypeus, labrum and anterolateral surfaces of first visible labial segment; on ventral portion of head (gula), several rows of shorter, more numerous, obliquely erect setae as a pubescence; antennal segments I and II covered with adpressed or obliquely semi-erect thin pale setae; on segment I, much longer fine (ciliated) setae, which are approximately three to four times as long as the transverse width of the segment (Fig. 4); segments III and IV covered with more numerous and shorter, adpressed, straight or slightly curved thin setae. ***Thorax***: dorsal portion mostly glabrous, except in the anterior and lateral portions of mesoscutum which are covered by setae; median portion of meso- and metasternum covered with numerous short, erect setae forming a pubescence. Legs: coxae and trochanters covered with thin, decumbent, pale setae; armature of inner face of left fore coxa with three spines, lined on the same direction, at submedian basal, submedian distal and apical positions (Fig. 5); right fore coxa with five spines, four lined at similar positions as the other coxa and other smaller spine more anteriorly located, at the level between the two more basal spines (Fig. 6); fore femora covered with numerous thin, long, obliquely erect setae and more abundant erect setae on ventral surface; among the latter, five setae are even longer, approximately as long as the width of the segment, straight and somewhat larger (Fig. 5); armature with seven spines on upper margin of inner surface (Figs 5, 7); ventrally, at approximately the basal third, two long spines preceded by one spine half as long as the others and very short spines along the approximately distal two thirds of the segment (Fig. 5); a subapical ventral protuberance shallow with three minute spines (Fig. 5); middle and hind femora, tibiae and tarsi generally covered with thin, short and long setae, which are even more numerous towards apical portion of tibiae. Armature of fore tibiae with a single longitudinal median row of 17 (left tibia) (Fig. 5) or 18 (right tibia) spines at inner face, beginning somewhat far from base and ending far from apex of the segment, running by a distance of approximately 1/2 of the length of the segment; the spines are somewhat larger basally, becoming smaller towards distal portion of the row; additionally, three small spines, slightly positioned anteriorly, intercalated with spines which form the row at approximately its distal half (Fig. 5). The distal portion of the fore tibiae generally more densely covered by numerous stout adpressed setae; on inner face, distally to the end of the rows, these are even more numerous and subapically, a small subapical pecten. Forewing mostly glabrous, with thin, long setae on the costal vein, basal portion of the area subjacent to the anal vein, and short sparse setae on the margins of the pterostigma. Hind wing glabrous. *Abdomen* covered by thin, pale setae, which are generally shorter on the tergites and longer on sternites. Ventral portion of distal third of pygophore covered by very long, thin and numerous setae (Figs 8–10); a tuft of setae anterior to the implantation of parameres (Figs 8–10); posterior surface of medial process of the pygophore with a few short erect setae (Figs 8, 10, 12).

**Figures 14–17.**
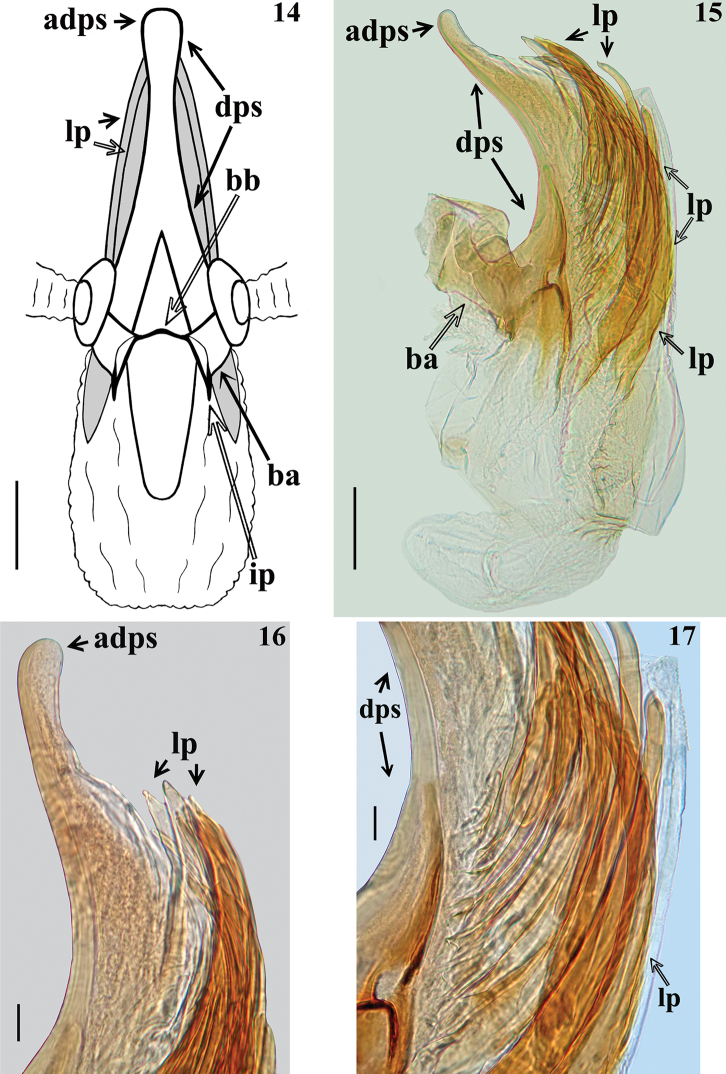
*Quasitagalis afonsoi* gen. et sp. nov., male holotype, phallus **14** dorsal view **15–17** lateral view **16** distal portion **17** median portion. Abbreviations: adps apex of dorsal phallothecal sclerite, ba basal arm, bb basal bridge, dps dorsal phallothecal sclerite, ip inferior prolongation of the basal arm, lp laminar process (es). Scale bars: 0.1 mm (**14, 15**); 0.02 mm (**16, 17**).

**Figures 18–20.**
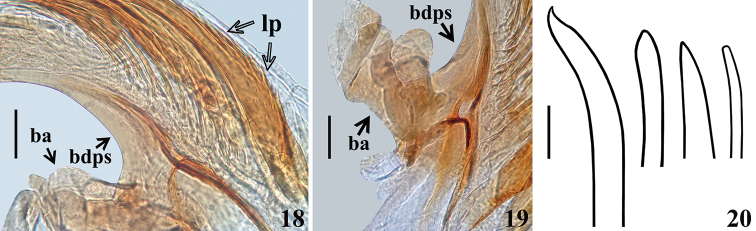
*Quasitagalis afonsoi* gen. et sp. nov., male holotype, phallus. **18, 19** Basal portion, lateral view **20** shape of apical portion of some laminar processes of endosoma. Abbreviations: ba basal arm, bdps basal portion of dorsal phallothecal sclerite, lp laminar process. Scale bars: 0.05 mm (**18, 19**); 0.02 mm (**20**).

***Structure.*** Venation of both wings similar to that of the female (Figs 34, 37). Segment VIII sclerotised on ventral portion, dorsal portion membranous; spiracles above dorsal margin of sclerotised ventral portion (Figs 8, 9). *Male genitalia* (Figs 8–20): genital capsule in situ (Figs 8, 9) with the apex of dorsal phallothecal sclerite (adps) prominent, just anterior to medial process of pygophore (mpp) and only the distal third of parameres (pa) visible, within pygophore rim. Pygophore in dorsal view (Fig. 11): somewhat elongated and ovoid in shape; between anterior and posterior genital openings, a dorsal (transverse) narrow bridge; margins of anterior opening subrounded; margins of posterior opening sinuous; in lateral view (Fig. 10): ventral margin rounded; dorsal margin rounded at approximately basal half and almost straight at distal half. Medial process of pygophore (mpp) (Figs 8, 10–12) narrow, somewhat elongated, curved in lateral view, tapering. Paramere (pa) (Figs 8, 9, 10, 13) short, strongly curved at approximately middle third, somewhat wider at distal two thirds; rounded at apex, in which a strong median apical acute spine is implanted in the same direction of the body of the paramere; glabrous at approximately basal two thirds and with sparse elongated somewhat curved thin setae scattered on distal third. Phallus (Figs 14–20): articulatory apparatus with short, stout basal arms (ba) (Figs 14, 15, 19), connected by a narrow basal bridge (bb) (Fig. 14); basal arms with a small, pointed inferior prolongation (Fig. 14, ip). Dorsal phallothecal sclerite (dps) (Figs 14–19) faintly sclerotised, elongated, curved in lateral view (Figs 15, 17); in dorsal view (Fig. 14): larger at basal portion, subtriangular at basal third, progressively narrowing towards middle third and somewhat enlarged at distal third, the latter surpassing the other elements of the phallus (Figs 15, 16); apical margin rounded (Figs 14–16). Ventrally to the dorsal phallothecal sclerite, numerous, elongated, sclerotised, curved, laminar processes (lp) (Figs 14–18, 20). These processes are larger at their bases, narrowing, not uniformly, towards their apices, which vary between presenting from a thin to a wider width as well as their tips which also vary as being rounded to acutely pointed (Fig. 20).

**Female.** Figures 21–51. Measurements are given in Table 1. Similar to male in general. The recorded differences in size are given in Table 1.

**Figures 21–24.**
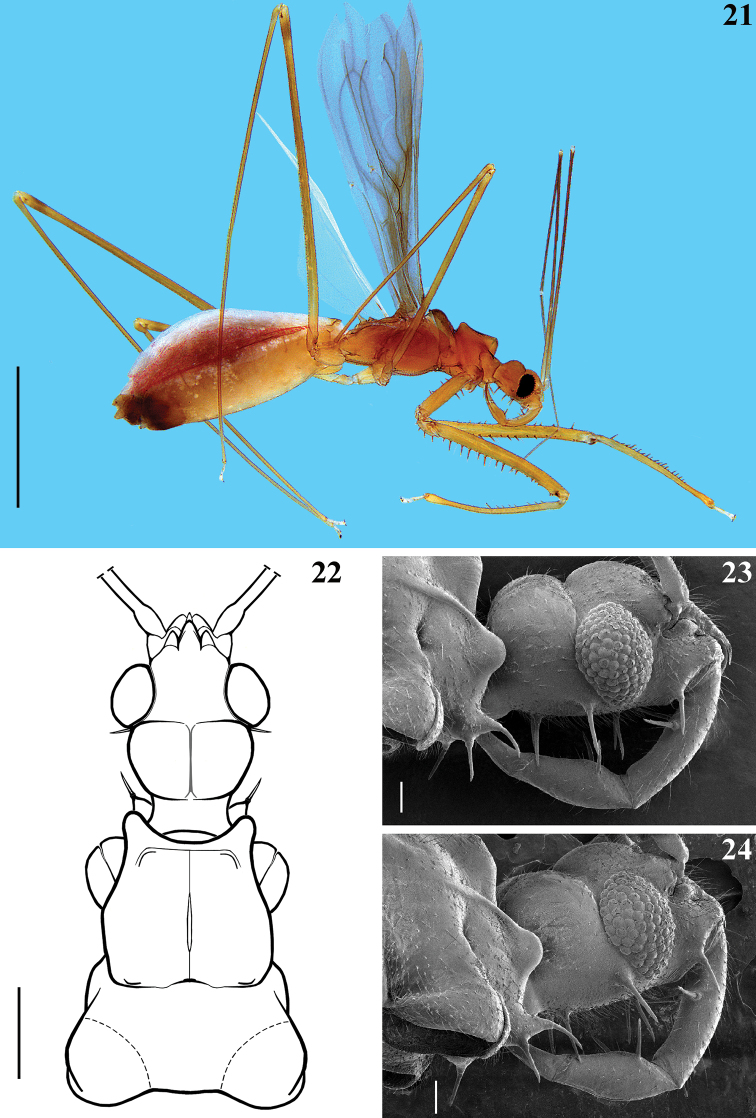
*Quasitagalis afonsoi* gen. et sp. nov., female paratypes **21** lateral view **22** head and pronotum, dorsal view **23, 24** head and anteroinferior portion of prothorax **23** lateral view **24** posterolateral view. Scale bars: 2.0 mm (**21**); 0.5 mm (**22**); 0.1 mm (**23, 24**).

**Figures 25–32.**
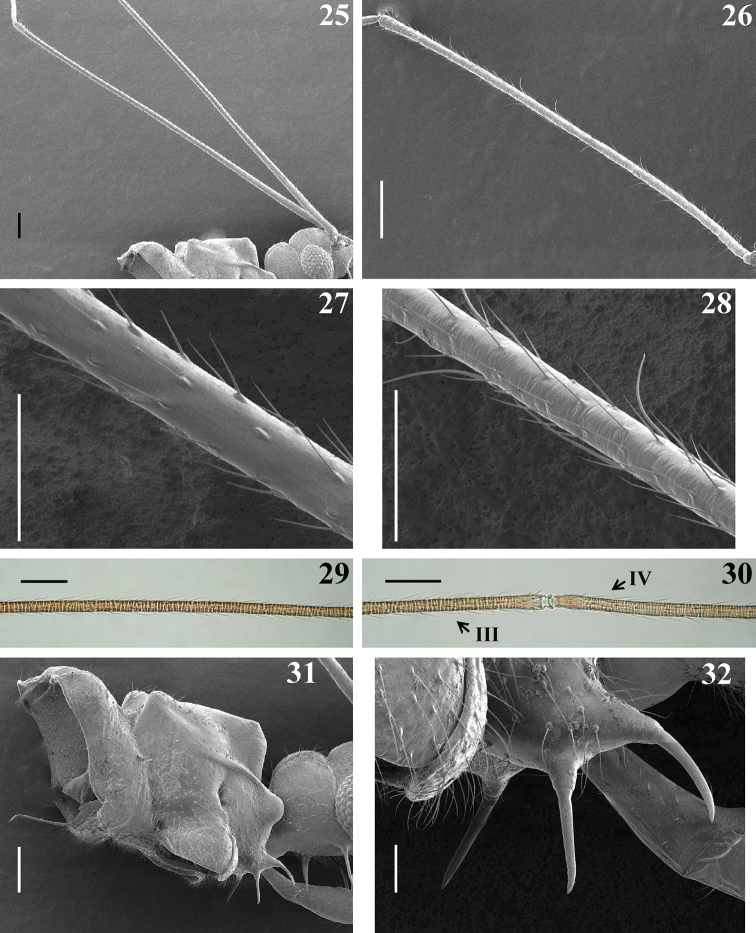
*Quasitagalis afonsoi* gen. et sp. nov., female paratypes **25** upper portion of head and pronotum and antennal segment I, lateral view **26** antennal segment II **27–29** median portion of antennal segments **27** segment I **28** segment II **29** segment III **30** distal and basal portions of antennal segments III and IV (III and IV, respectively) **31** posterior portion of head and prothorax, lateral view **32** proepisternal process, lateral view. Scale bars: 0.2 mm (**25, 26, 31**); 0.1 mm (**27–30**); 0.05 mm (**32**).

***Vestiture.*** first antennal segment without long ciliated setae (Figs 25, 27); armature of inner face of both coxae with three spines, lined in a same direction, at submedian basal, submedian distal and apical positions (Figs 38, 39, 42); armature of fore femora (Figs 38–40, 43–46) with nine (Figs 39, 44) to ten (Figs 38, 43) spines on upper margin of inner surface; between the latter and the ventral line of spines, an intermediate row with 19 spines, similar in size (Figs 38, 39, 43); ventrally, at approximately the basal two thirds, four to seven long spines intermixed with four or five shorter spines; at approximately distal third, four to eight shorter spines, similar in size or progressively smaller towards the apex of the segment (Figs 38, 39, 43, 45, 46). Armature of fore tibiae (Figs 38, 39, 48, 49) with a double row of numerous spines, the posterior row begins closer to the base of the segment and runs by approximately 2/3 along of the inner surface; the anterior row begins more distally, approximately posteriorly to the third or fifth posterior spine; both rows ending far from the apex; the spines are generally stronger than those recorded in the male; the approximately 11–15 anterior and 16 posterior spines are mostly implanted intercalated in relation to each other and the former are generally smaller than the latter (Figs 38, 39, 48, 49); in one specimen there were five or six small additional spines randomly distributed at the mid portion of the rows and the two or three distal spines of the posterior row are thinner than the other posterior spines.

**Figures 33–37.**
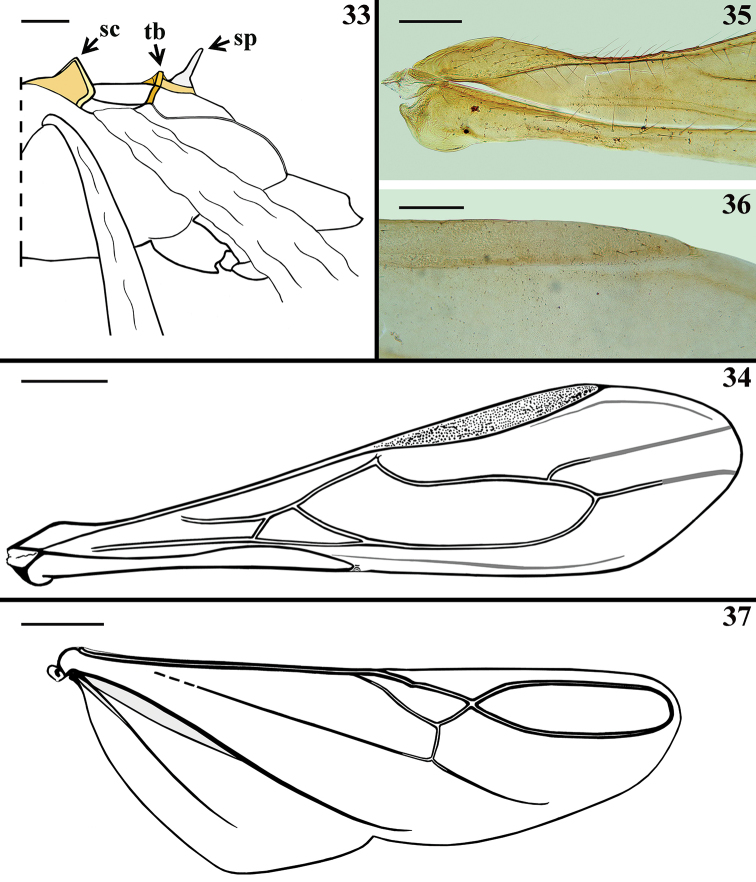
*Quasitagalis afonsoi* gen. et sp. nov., female paratypes **33** meso- and metathorax (wings moved down), lateral view **34–36** forewing **35** basal portion **36** apical half of pterostigma and subjacent portion **37** hind wing. Abbreviations: sc scutellum, sp spine of metanotum, tb tuberculum of metanotum. Scale bars: 0.5 mm (**34, 37**); 0.2 mm (**33, 35, 36**).

***Structure.*** Venation of both wings as shown in Figs 34, 37. Female genitalia: posterior view of external genitalia as in Fig. 51.

###### Distribution.

Brazil, State of Tocantins, Lavandeira municipality, Gruta da Gia [Gia’s Cave], 12°49'42"S, 46°20'43"W.

###### Etymology.

The new species is named in honour to Professor and Researcher Dr Luiz Afonso Vaz de Figueiredo for his role as an environmentalist, responsible for the training of countless educators; a great supporter of Speleology in Brazil.

###### Comments.

**Figures 38, 39.**
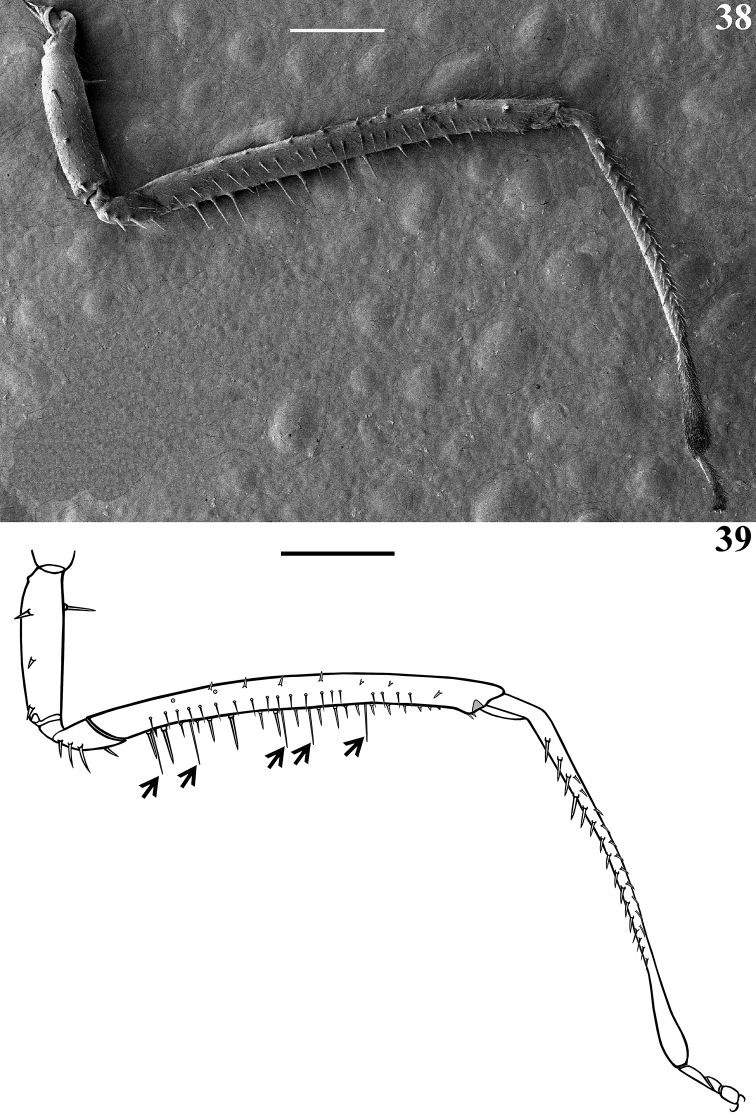
*Quasitagalis afonsoi* gen. et sp. nov., female paratypes **38, 39** fore leg, inner face, lateral view **39** setae omitted, except the longer setae of femur, pointed by arrows. Scale bars: 0.5 mm.

Among the two common sexual dimorphic characteristics recorded among Saicinae, such as in species of *Tagalis* (e.g., Gil-Santana et al. 2010, Gil-Santana 2011), the larger eyes and longer, ciliated setae on the first antennal segment of males, only the latter was undoubtedly recorded in the specimens of *Quasitagalis afonsoi* examined here (Fig. 4). The eyes of the male holotype showed the same measurements as one female and did not seem much larger (Figs 1–3, 21–23) as it was recorded in males of some species of *Tagalis* (e.g., Gil-Santana et al. 2010; Gil-Santana 2011; Castro-Huertas and Forero 2014), but not in *Tagalis i. inornata* in which, the male eyes were shown not to be much larger than those of the females (Gil-Santana 2011). On the other hand, the armature of the fore femora of the females had more spines in general, including an additional row of spines (intermediate between those of upper margin and ventral margin of inner face) (e.g., Figs 5, 38, 39). Yet, in the females, the armature of the fore tibiae was more prominent, formed by a double row of much stronger spines, extending comparatively for a longer distance on the inner surface (Figs 38, 39, 48, 49), while in the male holotype, although there were three small, anterior spines analogous to that of the anterior row of the female, the armature was basically formed by a single row of generally smaller spines, extending for a shorter distance on the inner surface of its tibiae (Fig. 5). Similarly, for *Tagalis*, McAtee and Malloch (1923) argued that the spines on the fore femora were sexually dimorphic, while the armature of the fore femora showed smaller spines in the males of *Tagalis i. inornata* than in the females of this species examined by Gil-Santana (2011). However, both the confirmation that the eyes are of similar size in both sexes as well as if the differences in the armature of the fore legs are part of sexual dimorphism or a part of individual variation in *Quasitagalis afonsoi* would be better evaluated through examining more specimens.

**Figures 40–47.**
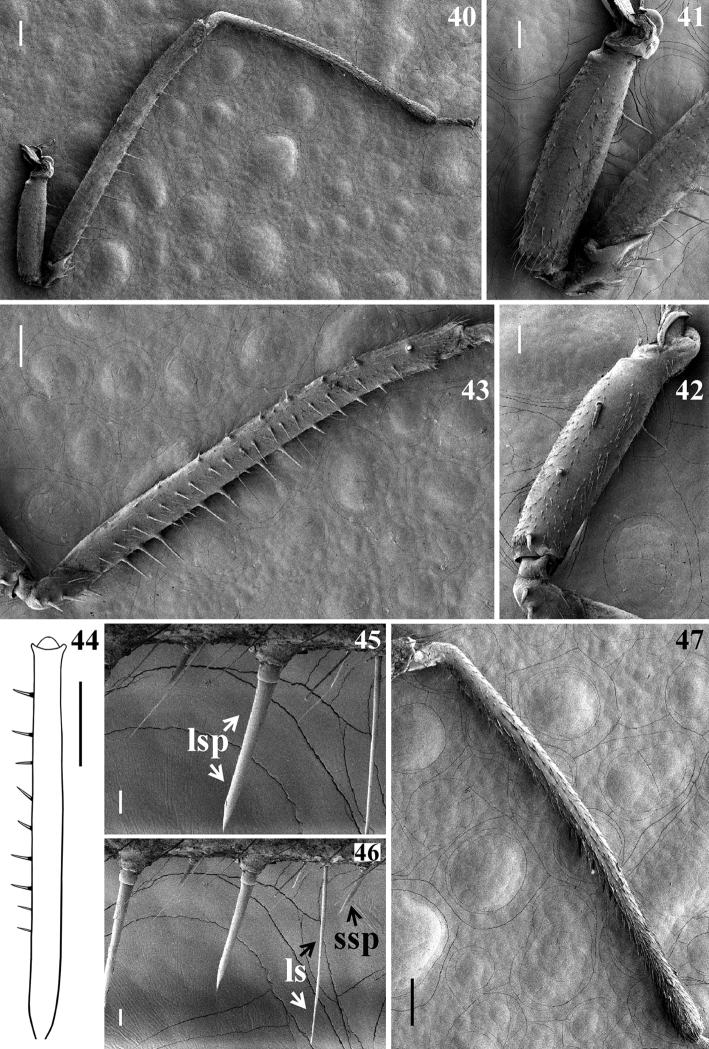
*Quasitagalis afonsoi* gen. et sp. nov., female paratypes, fore leg **40–43, 45–47** lateral view **40, 41** outer face **41** coxa, trochanter and base of femur **42, 43** inner face **42** coxa and basal portion of trochanter **43** trochanter and femur **44** right femur, dorsal view **45, 46** segment of ventral portion of femur **47** tibia, outer face. Abbreviations: ls longer seta, lsp long spine, ssp short spine. Scale bars: 0.5 mm (**44**); 0.2 mm (**40, 43, 45–47**); 0.1 mm (**41, 42**).

**Figures 48–51.**
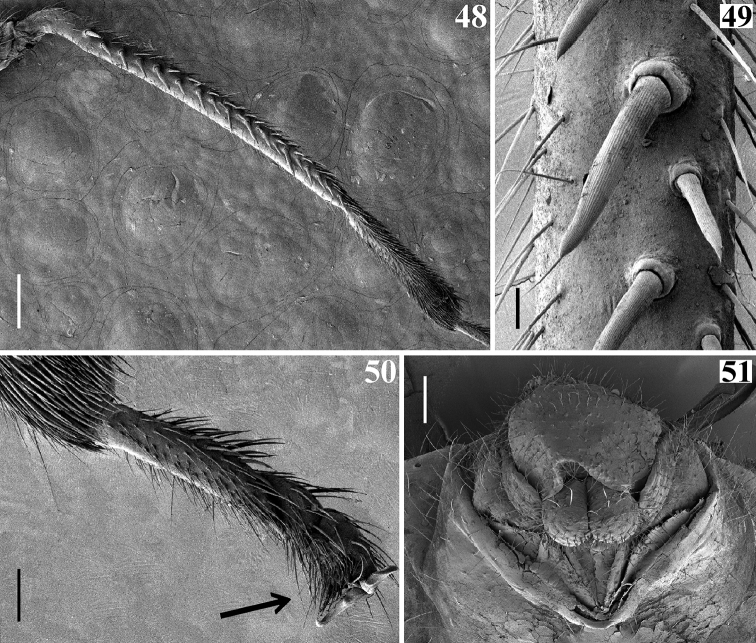
*Quasitagalis afonsoi* gen. et sp. nov., female paratypes **48, 49** fore tibia, lateral view, inner face **49** portion of the double row of spines; at the center a pair of the latter in which the posterior spine is larger and longer than the anterior one **50** fore tarsus, ventral view, the arrow points to the scopula **51** genitalia, posterior view. Scale bars: 0.2 mm (**48**); 0.1 mm (**51**); 0.05 mm (**50**); 0.02 mm (**49**).

###### Additional material examined.

*Quasitagalis afonsoi* gen. et sp. nov. **Brazil**, Tocantins, Lavandeira, Gruta da Gia [Gia’s Cave], 05–10.i.2009, 12°49'42"S, 46°20'43"W, Robson A. Zampaulo leg., 2 nymphs (CTJMSB, 861). *Tagalis inornata inornata* Stål, 1860. **Brazil**, Rio de Janeiro, Nova Friburgo, 22°17'S, 42°29'W, 1.049 m, 01 female, 09.xii.1997 (CTJMSB, 862).

###### Remarks on nymphs.

**Figures 52–54.**
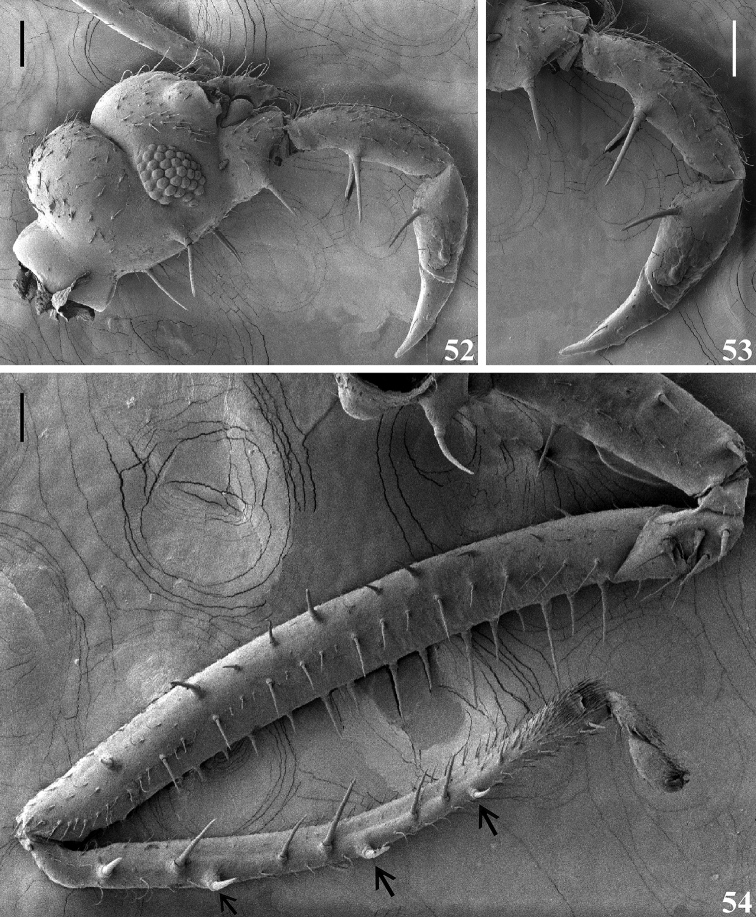
*Quasitagalis afonsoi* gen. et sp. nov., earlier instar nymph **52–54** lateral view **52** head **53** labium **54** fore leg, inner face, the setae point to the larger setigerous spines on inner surface of fore tibiae, close to its dorsal surface. Scale bars: 0.1 mm.

**Figures 55–59.**
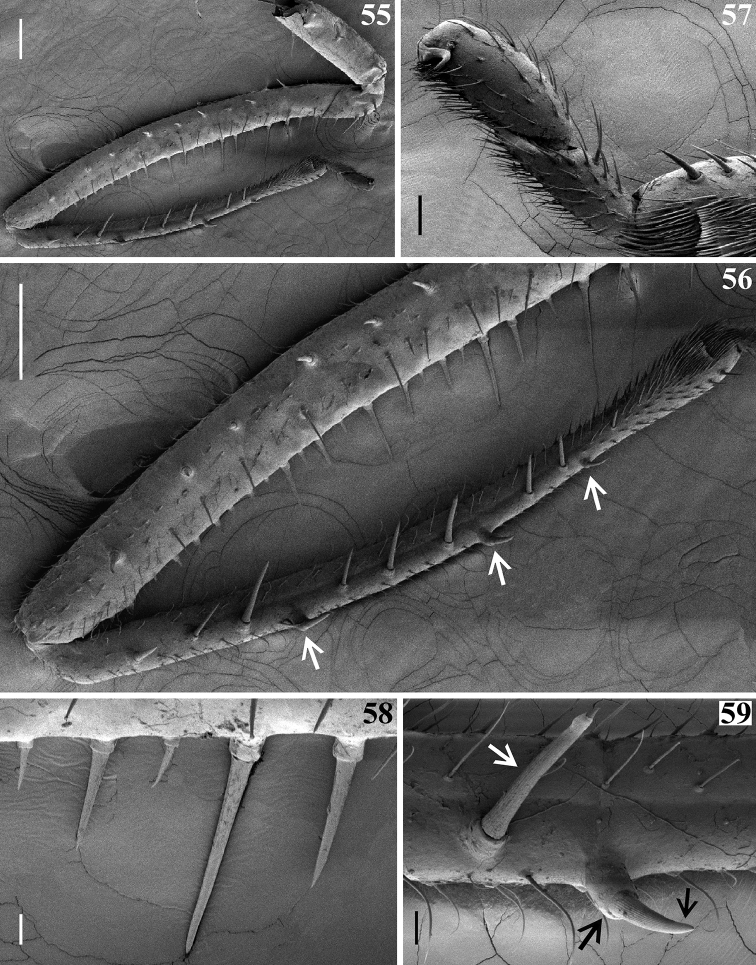
*Quasitagalis afonsoi* gen. et sp. nov., later instar nymph, fore leg, lateral view **55, 56** inner face **55** entire leg **56** approximately distal two thirds of femur and tibia; the setae point to the larger setigerous spines on inner surface of fore tibiae, close to its dorsal surface **57** tarsus **58** basal portion of the ventral armature of femur **59** portion of tibia in which the second lateral spine (pointed by black arrows) is inserted; adjacent long submedian spine pointed by a white arrow. Scale bars: 0.2 mm (**55, 56**); 0.04 mm (**57**) 0.02 mm (**58, 59**).

Two nymphs of *Quasitagalis afonsoi* were collected with the adults. They were from different and undetermined instars and were not in good condition for formal or complete descriptions. However, the head of the smaller (earlier instar) nymph and fore legs of both of them were well conserved and were used to obtain SEM images (Figs 52–59). Their measurements are given in Table 2.

**Table 2.** Measurements (mm) of nymph specimens of *Quasitagalis afonsoi* gen. et sp. nov.

	Earlier instar nymph	Later instar nymph
Body length to tip of abdomen	3.50	4.90
Head length (excluding neck)	0.50	0.60
Anteocular portion length	0.20	0.20
Postocular portion length	0.25	0.30
Head width across eyes	0.40	0.50
Interocular distance (synthlipsis)	0.30	0.30
Transverse width of eye	0.05	0.10
Length of eye	0.10	0.15
Antennal segment I length	2.10	2.60
Antennal segment II length	1.00	1.40
Antennal segment III length	1.20	1.50
Antennal segment IV length	0.90	1.00
Labial segment II length	0.35	0.40
Labial segment III length	0.20	0.30
Labial segment IV length	0.15	0.20
Pronotum length	0.50	0.60
Pronotum maximum width	0.60	0.60
Wing pad length	0.70	1.30
Fore coxa length	0.50	0.60
Fore femur length	1.40	1.70
Fore tibia length	1.20	1.50
Fore tarsus length	0.25	0.25
Mid femur length	1.80	2.40
Mid tibia length	2.30	3.10
Mid tarsus length	0.20	0.20
Hind femur length	absent	3.40
Hind tibia length	absent	4.20
Hind tarsus length	absent	0.20
Abdomen length	1.00	2.50
Abdomen maximum width	0.40	0.80

They are shown to have bi-segmented tarsi (Figs 54, 57) and smaller eyes (Fig. 52), common features found in immature Heteroptera (Schuh and Weirauch 2020; Rédei 2004) and also in the nymphs of *Tagalis evavilmae* (Gil-Santana et al. 2010). The armature of the head is like those recorded in the adults, i.e., with strong setigerous spines anteroventrally and posteroventrally from eyes, the former somewhat smaller than the latter and ventrally, on the gula, two pairs of similar setigerous spines: one pair below (between) the eyes and other pair, posterior to the eyes, somewhat closer to the neck than to eyes (Fig. 52); labial segments II and III with a pair of stout spines, slightly basal to midpoint and on swollen portion, respectively (Figs 52, 53). The structure and armature of the fore coxa is also like those of the adults in general (a long spine on basal third of anterior surface and three spines on its inner surface) (Figs 54, 55). The armature of the fore trochanter is similar, but with small differences: the apical (fourth) spine is comparatively smaller and there is an additional spine below the third spine in the nymph of earlier instar (Fig. 54). The armature of the fore femora is equivalent to that of the adult female (Figs 54–56, 58): armed ventrally with a few short spines and a variable number of few longer ones intermixed at approximately the basal two thirds and a small subapical ventral protuberance with small spines; a row of spines on the upper margin of the inner surface, which are comparatively longer than those of adults (with ten elements in the earlier instar nymph and nine in the later instar nymph); between the latter and the ventral line of spines, a row of setigerous spines, more variably in size, with approximately 14 elements in both nymphs. The armature of the fore tibia, however, is conspicuously different from that of the adults, with a submedian (somewhat lateral to the median portion of the segment) row of six (earlier instar nymph) (Fig. 54) or eight (later instar nymph) (Figs 55, 56) strong, long spines on the inner surface (besides the more distal spine in both nymphs which is shorter and smaller); at the lateral border of the inner surface, three larger but shorter setigerous spines (Figs 54–56); at the apical portion of the dorsal surface, two or three small curved spines (Figs 54, 56, 57).

###### Habitat.

**Figures 60–63.**
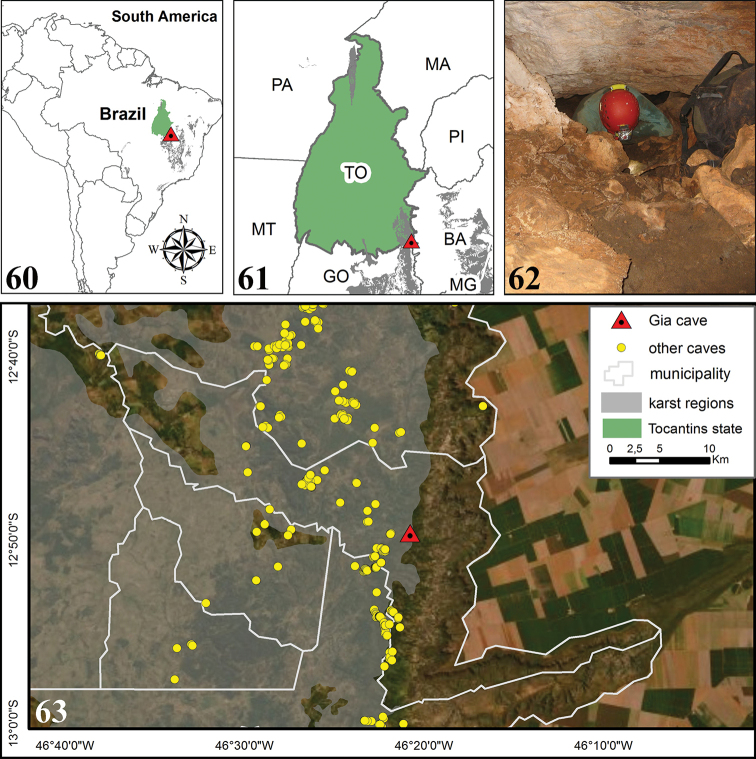
**60, 61** Position of the Gruta da Gia [Gia’s Cave], the type locality of *Quasitagalis afonsoi* sp. nov., in relation to Brazil and the State of Tocantins **62** the entrance to Gruta da Gia [Gia’s Cave] **63** Position of the Gruta da Gia [Gia’s Cave] in relation to the municipality of Lavandeira, other caves identified in the region and the delimitation of carbonate rocks (the Bambuí Geological Group in light gray). Abbreviations: Brazilian States: BA Bahia, GO Goiás, MA Maranhão, MG Minas Gerais, MT Mato Grosso, PA Pará, PI Piauí, TO Tocantins.

The municipality of Lavandeira is located at 12°47'19"S, 46°24'28"W, in the southeast of the State of Tocantins and Northern Brazil (Figs 60, 61, 63) with an altitude of approximately 330 meters and an area of 519,614 km². The climate in the region is tropical with average annual rainfall ranging from 1,400 to 1,600 mm and average temperature ranging from 25 to 27 °C (SEPLAN 2008). The predominant vegetation is the Cerrado (Brazilian savannas), covering 87.8 % of the state’s area, the rest is occupied by forests. It is noteworthy that the Cerrado is considered one of the main biodiversity hotspots (priority areas for biodiversity conservation) worldwide (Myers et al. 2000; Mittermeier et al. 2011; Williams et al. 2011).

The carbonates present in the region are located in the Speleological Province of the Bambuí Group (São Domingos District) and distributed in a north-south direction. At this portion, elevations vary between 400 and 600 m, while elevations below 400 m dominate the northwestern portion of the study area. The Bambuí Group constitutes the largest set of limestone occurrences, favourable to the presence of caves in Brazil (Karmann and Sánchez 1979).

Currently, the State of Tocantins has 939 caves registered in the official government databases (CECAV 2019), with approximately 350 known caves in the southeastern region. However, karst in the region has the potential for thousands of caves. Gruta da Gia [Gia’s Cave] is located in the central portion of the municipality of Lavandeira (Fig. 63) under the 12°49'42"S, 46°20'43"W at 503 m high. Inserted at the top of the limestone massif, this cave is approximately 200 m of long and has only one entrance (Fig. 62). It is a humid cave with clay soil, extensive aphotic zones and low availability of trophic resources. The surrounding landscape is made up of pasture areas, but there are still extensive fragments of native forest associated with karst outcrops and springs areas. In general, the caves in the region have small dimensions, the majority of which are less than 50 m long. They have a predominantly flat floor, and few have underground lakes or rivers.

**Figures 64, 65.**
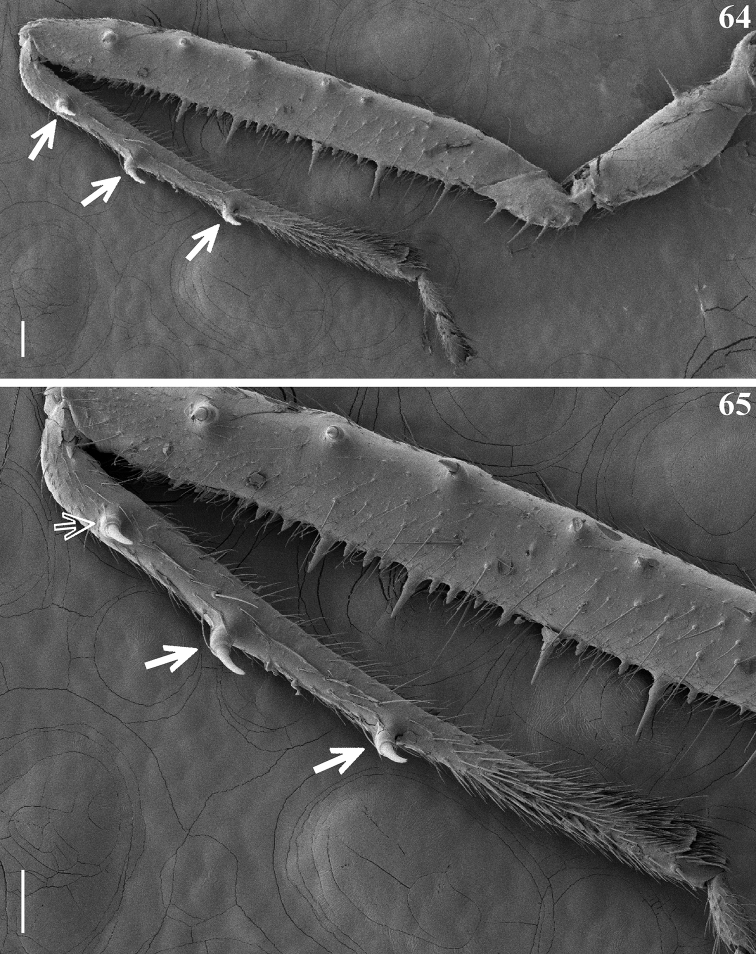
*Tagalis inornata inornata* Stål, 1860, female, fore leg, lateral view, inner face; the setae point to the larger setigerous spines on inner surface of fore tibiae, close to its dorsal surface **64** entire leg **65** portion of femur and tibia. Scale bars: 0.1 mm.

## Discussion

Gil-Santana et al. (2010) argued that the revised description of *Tagalis* when compared to the one of *Paratagalis* Monte, 1943 (Gil-Santana and Costa 2009) showed many similarities, suggesting that they were closely related genera, what was reinforced by Gil-Santana (2011), while studying additional species of *Tagalis*. A similar situation is revealed in relation to *Tagalis* and *Quasitagalis*, both sharing many common characteristics, suggesting that they are also closely related genera.

It is noteworthy that, while *Paratagalis* and *Quasitagalis* have in common the presence of two pairs of setigerous spines on the gula (Figs 3, 23, 24), all other differential characteristics of the latter genus presented in relation to *Tagalis*, are also valid as such in relation to *Paratagalis*. On the other hand, some additional characteristics of *Quasitagalis afonsoi* sp. nov. were not recorded in any species of *Paratagalis* or *Tagalis* so far. Those are the additional row of spines (intermediate between those of the upper margin and ventral margin of the inner face) of the fore femora in the females (Figs 38, 39, 43) and the shape of parameres in the male (Fig. 13).

Regarding the male genitalia, it is noteworthy that the shape of the pygophore, its process, the endosoma portions (articulatory apparatus, dorsal phallothecal sclerite, curved elongated processes of endosoma) (Figs 10–12, 14–20) have many similarities to what was recorded in other species of *Tagalis* (e.g., Gil-Santana et al. 2010; Gil-Santana 2011). However, while in some species of the latter genus, the pygophore showed a conspicuous lateral apophysis, it was not recorded in *Q. afonsoi*.

More importantly, in all species of *Paratagalis* and *Tagalis* in which the paramere was described (Blinn 2008; Melo 2008; Gil-Santana and Costa 2009; Gil-Santana et al. 2010; Gil-Santana 2011; Castro-Huertas and Forero 2014; Varela and Melo 2017), it showed to be elongated, strongly curved apically, or with an apical large teeth, which was implanted laterally, forming a right to an acute angle with the body of the paramere, while in *Q. afonsoi*, the paramere is short, rounded at apex, in which the strong apical acute spine is implanted in the same direction of the body of the paramere (Fig. 13).

The presence of the scopula on the apex of the third tarsomere on all the legs in *Q. afonsoi* (Fig. 50) is in accordance with the statements of Weirauch and Forero (2007a) and Weirauch (2008a) that this character seems to be widespread among Saicinae and is a synapomorphy of part of this subfamily, respectively. As such, it has been recorded in species belonging to several genera of Saicinae (Weirauch 2007; Weirauch and Forero 2007a, b), including two species of *Tagalis* (Castro-Huertas and Forero 2014), genus which seems to be closer to *Quasitagalis*

The three larger lateral setigerous spines on the inner surface of the fore tibiae, close to its dorsal surface, present in the nymphs of *Q. afonsoi* (Figs 54–56, 59) are striking, because they are very similar to those recorded in almost all species of *Tagalis* (Figs 64, 65) (*T. femorata* Melo, 2008 has four spines) and also in *Paratagalis* (Gil-Santana and Costa 2009), while they are completely absent in the adults of the new species (Figs 5, 38, 39, 48, 49). Similarly, Gil-Santana et al. (2010) recorded features in the nymphs of *T. evavilmae* not present in adults of *Tagalis* but observed in adults of other related genera, what they argued might help to understand the relationships among the genera of Saicinae in future studies. It is noteworthy that the mentioned features in the nymphs of *T. evavilmae* included two pairs of spines on the ventral side of the head as present both in adults and nymphs of *Q. afonsoi* sp. nov. (Fig. 52) and five (*T. evavilmae*) to six (*Q. afonsoi*) (Fig. 54) strong long spines on the inner face of the fore tibiae recorded in the nymphs of earlier stage of both species.

Future phylogenetic analyses should be carried out to assess all these similarities and differences and the taxonomic validity of the genera and their systematic positions, and the relationships among species of New World genera of Saicinae.

Several species of Emesinae, a group considered as close related to Saicinae (e.g., Wygodzinsky 1966; Weirauch 2008a; Schuh and Weirauch 2020), are occasionally found or consistently live in caves (Wygodzinsky 1966; Pape 2013; Gil-Santana and Ferreira 2017). On the other hand, as far as it seems, this is the first record of a species of Saicinae, at least in the New World, found inside or as a possible inhabitant of caves. The presence of a male, females and immature forms make believe that the presence of *Q. afonsoi* inside the cave was not an incidental finding, but more probably their life cycle was, at least, partially being carried out there, possibly the species might be reproducing in such habitats, as it occurs with some emesines (Wygodzinsky 1966; Pape 2013). However, more than a dozen caves were sampled in the region and few specimens were found in only one of them (Gia’s Cave), which gives this species a certain rarity. In any case, a more extensive study would be necessary, including more frequent collecting and searches around the cave in the epigean environment to determine the importance of caves as a habitat for *Q. afonsoi*. On the other hand, for similar reasons, and also because of the fact that part of the surrounding landscape is made up of pasture areas, which tend to extend, the distribution of the new species was considered here strictly restricted to the Gia´s Cave (Figs 62, 63), which should be also considered its type locality.

### Key to the New World genera of Saicinae

Based on Weirauch and Forero (2007a, b), Gil-Santana and Costa (2009), and Gil-Santana et al. (2015)

**Table:** 

1	Foreleg without spines, at most with erect setae	**2**
–	Fore femur with two or three rows of spines, fore tibiae either with setae or with spines	**5**
2	Posterior pronotal lobe with upward projecting spines or tubercles; mesoscutum (scutellum) and metanotum apically with vertical spines or tubercles	**3**
–	Pronotum generally unarmed, but sometimes with humeral spines; apex of mesoscutum produced into a long horizontal tapering spine, metanotum without spine or tubercle	***Oncerotrachelus* Stål, 1868**
3	Opposed surfaces of labium and head with spine-like setae or bristles; forewing with two to three cells; metapleura without a tubercle near coxal cavity	**4**
–	Opposed surfaces of labium and head without spine-like setae or bristles; forewing with four cells; metapleura with a tubercle near coxal cavity	***Saicireta* Melo & Coscarón, 2005**
4	Process on lower anterior angle of prothorax acute to subacute; second antennal segment approximately half as long as first antennal segment; medial process of male pygophore bifurcate; posterior margin of seventh abdominal sternite in females vertical or subvertical	***Saica* Amyot & Serville, 1843**
–	Process on lower anterior angle of prothorax subconical; second antennal segment approximately 3/4 as long as first antennal segment; medial process of male pygophore a single, erect spine; posterior margin of seventh abdominal sternite in females sloping ventrocephalad	***Pseudosaica* Blinn, 1990**
5	Humeral angles of pronotum without processes, rounded	**6**
–	Humeral angles of pronotum with spine-like processes	**7**
6	Ventral portion of the head below (between) the eyes spineless; fore tibiae with a three or four (*T. femorata*) stronger, setigerous spines implanted on external border of inner surface, close to dorsal surface	***Tagalis* Stål, 1860**
–	Head with a ventral pair of spines below (between) the eyes; fore tibiae with a single or double longitudinal row of numerous short spines on median portion of inner surface	***Quasitagalis* gen. nov.**
7	Fore coxae and anterior pronotal lobe unarmed	***Bagriella* McAtee & Malloch, 1923**
–	Fore coxae spined, anterior pronotal lobe with four spines or rounded humps	**8**
8	Fore lobe of pronotum with four spines	***Paratagalis* Monte, 1943**
–	Fore lobe of pronotum with four humps	**9**
9	Two first (visible) labial segments spiny; only apterous females known	***Kiskeyana* Weirauch & Forero, 2007**
–	Only the first (visible) or all three labial segments spiny; females macropterous	**10**
10	Only the first (visible) labial segment spiny; forewings with four closed cells	***Buninotus* Maldonado, 1981**
–	All three (visible) labial segments spiny; forewings with two closed cells	***Caprilesia* Gil-Santana, Marques & Costa, 2006**

## Supplementary Material

XML Treatment for Quasitagalis

XML Treatment for Quasitagalis afonsoi
